# Supplementary material for: Venous leg ulcer healing as a determinant of quality of life in patients treated with unna boot: A quasi-experimental study
Source: PLoS One. 2025 Jan 29;20(1):e0314490. doi: 10.1371/journal.pone.0314490 (PMC11778712; doi:10.1371/journal.pone.0314490)
Supplement: Attachment — Submitted filename: Response to Reviewers.pdf [file pone.0314490.s001.pdf]

Response to Reviewers

Dear Reviewers,

On behalf of the authors, I would like to extend my greetings and express our gratitude for the thoughtful comments and requests regarding the submitted manuscript. I am confident that they have helped improve the quality of the work to which we have been so dedicated.

Below, I have provided point-by-point responses to each of your comments. I hope these answers satisfactorily address the concerns you raised.

Comments to the Author

1. Is the manuscript technically sound, and do the data support the conclusions?

The manuscript must describe a technically sound piece of scientific research with data that supports the conclusions. Experiments must have been conducted rigorously, with appropriate controls, replication, and sample sizes. The conclusions must be drawn appropriately based on the data presented.

Reviewer #1: Yes

Reviewer #2: Yes

---

2. Has the statistical analysis been performed appropriately and rigorously?

Reviewer #1: Yes

Reviewer #2: Yes

---

3. Have the authors made all data underlying the findings in their manuscript fully available?

The PLOS Data policy requires authors to make all data underlying the findings described in their manuscript fully available without restriction, with rare exception (please refer to the Data Availability Statement in the manuscript PDF file). The data should be provided as part of the manuscript or its supporting information, or deposited to a public repository. For example, in addition to summary statistics, the data points behind means, medians and variance measures should be available. If there are restrictions on publicly sharing data—e.g. participant privacy or use of data from a third party—those must be specified.

Reviewer #1: Yes

Reviewer #2: No

---

4. Is the manuscript presented in an intelligible fashion and written in standard English?

PLOS ONE does not copyedit accepted manuscripts, so the language in submitted articles must be clear, correct, and unambiguous. Any typographical or grammatical errors should be corrected at revision, so please note any specific errors here.

Reviewer #1: No

Reviewer #2: No

---

5. Review Comments to the Author

Please use the space provided to explain your answers to the questions above. You may also include additional comments for the author, including concerns about dual publication, research ethics, or publication ethics. (Please upload your review as an attachment if it exceeds 20,000 characters)

Reviewer #1:

Dear Authors

Thank you for the opportunity to read the research results. I suggest making the following corrections/additions:
- in Introduction - population 1% - outdated data ...

Response: We found a meta-analysis published in 2023 that reports a prevalence of 0.32% and an incidence of 0.17%. We have replaced the theoretical reference accordingly. The study is described as follows:
Probst, S., Saini, C., Gschwind, G., Stefanelli, A., Bobbink, P., Pugliese, M. T., Cekic, S., Pastor, D., & Gethin, G. (2023). Prevalence and incidence of venous leg ulcers—A systematic review and meta-analysis. *International Wound Journal, 20*(9), 3906–3921. https://doi.org/10.1111/iwj.14272

- during treatment - how was the progress of ulcer healing assessed? what tool...?

Response: At each dressing change appointment at the service, a new evaluation was conducted by the healthcare professional. The evaluation focused on the same aspects, including the size and width of the wound, the appearance of its edges and interior, as well as the presence and characteristics of exudate. We have added this information to the methodology to make it clearer.

- inclusions criteria - only age? nothing else?, gender, other data?

Response: In addition to age as a requirement, the participant had to be registered with the primary care service in the study setting and not with other services. Furthermore, the venous ulcer had to be considered active, meaning it should not have healed. Even when covered by thin layers of skin, a wound could still be confused with an active venous ulcer. We have added a clarification in the methods section to better explain this point.

- what were the recommendations for patients undergoing compression therapy treatment (we have 1 year of observation here)

Response: Patients were routinely instructed on how to care for their dressings at home, such as protecting them during bathing to avoid getting them wet, following a healthy diet, and elevating the leg above heart level. All these instructions are in line with the conduct manual adopted as a theoretical reference cited in the manuscript. We have added a description of these instructions in the methodology to make it clearer.

- what treatment was provided apart from Unna boot? (pharmacology, dressings?()

Response: The treatment primarily consisted of an Unna boot covered by a secondary dressing (over the boot). Only in cases suggestive of wound infection did the physician implement antibiotic treatment before starting compression therapy. This is because infectious processes contraindicate the use of compression therapy. We have described this in the methodology.

- I suggest English Native Speaker correction

Response: We conducted a thorough revision of the English language with the assistance of a native speaker. The revision was both grammatical, and we aimed to make the language more fluid for better comprehension.

Reviewer #2:

Dear Author,
The manuscript is technically sound piece of scientific research with data that supports the conclusions. Clinical study has been conducted rigorously, with appropriate controls, replication, and sample sizes. statistical analysis is adequate and optimum. Data is presented well. English and grammar needs improvement.
Thanks
With Best Regards

Response: Thank you for your comments and observations. We have conducted a thorough revision of the English language with the help of a native speaker. The revision focused on grammar, and we have worked to improve the fluidity of the language for better understanding.
